# Downregulation of L1 perturbs neuronal migration and alters the expression of transcription factors in murine neocortex

**DOI:** 10.1002/jnr.23141

**Published:** 2012-10-17

**Authors:** Tomokazu Kishimoto, Kyoko Itoh, Masafumi Umekage, Madoka Tonosaki, Takeshi Yaoi, Kenji Fukui, Vance P Lemmon, Shinji Fushiki

**Affiliations:** 1 Department of Pathology and Applied Neurobiology, Graduate School of Medical Science, Kyoto Prefectural University of MedicineKyoto, Japan; 2 Department of Psychiatry, Graduate School of Medical Science, Kyoto Prefectural University of MedicineKyoto, Japan; 3 The Miami Project to Cure Paralysis, University of Miami School of Medicine, Lois Pope LIFE CenterMuiamio, Florida

**Keywords:** L1cam, cortex, migration, shRNA, in utero electroporation

## Abstract

L1 is a cell adhesion molecule associated with a spectrum of human neurological diseases, the most well-known being X-linked hydrocephalus. L1 knockout (L1-KO) mice have revealed a variety of functions of L1 that were crucial in brain development in different brain regions. However; the function of L1 in neuronal migration during cortical histogenesis remains to be clarified. We therefore investigated the corticogenesis of mouse embryos in which L1 molecules were knocked down in selected neurons, by employing in utero electroporation with shRNAs targeting L1 (L1 shRNA). Although more than 50% of the cells transfected with no small hairpin RNA (shRNA; monster green fluorescent protein: MGFP only) vector at embryonic day 13 (E13) reached the cortical plate at E16, significantly fewer (27%) cells transfected with L1 shRNA migrated to the same extent. At E17, 22% of cells transfected with the MGFP-only vector were found in the intermediate zone, and significantly more (34%) cells transfected with L1 shRNA remained in the same zone. Furthermore, the directions of the leading process of neurons transfected with L1 shRNA became more dispersed compared with cells with the MGFP-only vector. In addition, two transcription factors expressed in the neurons, Satb2 and Tbr1, were shown to be reduced or aberrantly expressed in neurons transfected with L1 shRNA. These observations suggest that L1 plays an important role in regulating the locomotion and orientation of migrating neurons and the expression of transcription factors during neocortical development that might partially be responsible for the abnormal tract formation seen in L1-KO mice. © 2012 Wiley Periodicals, Inc.

The neural cell adhesion molecule L1 is one of the most intensively studied adhesion molecules expressed in the developing central and peripheral nervous systems (Lemmon et al.,^1989^; Kamiguchi et al.,1998a, b; Kamiguchi and Lemmon,^1998^). L1 molecules play important roles in neuronal migration; axonal growth, guidance, and fasciculation; and neuronal survival and synaptic plasticity (Lemmon et al.,^1989^; Kamiguchi et al.,1998a). L1, a member of the immunoglobulin superfamily, is an integral membrane protein with six immunoglobulin (Ig)-like domains at the amino terminal end, followed by five fibronectin type III homologous repeats, a single transmembrane region, and a highly conserved cytoplasmic tail. L1 binds to a number of extracellular partners such as the proteoglycan neurocan neuropilin, α1β3 integrins, axonin-1/TAG-1, and contactin/F3/F11, as well as to itself in a homophilic manner (Ruppert et al.,^1995^; Kunz et al.,^1996^; Montogomery et al.,^1996^; Kamiguchi et al.,1998a; Yip et al.,^1998^; Kenwrick et al.,^2000^; Oleszewski et al.,^2000^; Silletti et al.,^2000^; Jacob et al.,^2002^). The heterophilic and homophilic interactions between L1 molecules and various ligands are thought to be required for axonal growth and pathfinding, migration, and neuronal survival during brain development.

Several X-linked human neurological diseases with links to mutations in the L1 gene have been reported, including X-linked hydrocephalus, MASA syndrome (mental retardation, aphasia, shuffling gait, adducted thumbs), agenesis/dysgenesis of the corpus callosum, and X-linked spastic paraplegia and CRASH syndrome (Yamasaki et al.,^1997^; Kamiguchi et al.,^1998^b,c; Weller and Gartner,^2001^). The phenotype common to these diseases is congenital hydrocephalus; however, the underlying mechanisms remain to be elucidated. It has been reported that L1-knockout (L1-KO) mice showed a phenotype strikingly similar to that of human patients with L1 mutations, including congenital hydrocephalus; hypoplasia of cerebellar vermis; abnormal corticospinal, thalamocortical, and retinotectal tracts; agenesis of corpus callosum; and abnormal hippocampal development (Dahme et al.,^1997^; Cohen et al.,^1998^; Fransen et al.,^1998^; Kamiguchi et al.,1998b; Demyanenko et al.,^1999^). These findings suggest that L1 is involved in various critical phases of brain development and should have different functional roles in different brain regions.

To assess which of the L1 interactions underlie the phenotypes observed in the L1-KO mice, a knock-in mouse in which the sixth Ig domain of L1 was deleted (L1-6D; Itoh et al.,^2004^, ^2005^) was examined. It was expected that this deletion would prevent L1-L1 homophilic binding and L1 binding to RGD-dependent integrins but that it would not disrupt interactions with neurocan or neuropilin. The L1-6D mutant mice seldom displayed hydrocephalus on the 129/Sv background. However, the same L1-6D mutation produced severe hydrocephalus on the C57BL/6J background, similarly to conventional L1-KO mice (Dahme et al.,^1997^). Recently, linkage studies utilizing χ^2^ tests and quantitative trait loci mapping confirmed a candidate modifier loci for a locus on chromosome 5, which we named *L1 hydrocephalus modifier 1* (Tapanes-Castillo et al.,^2009^).

To evaluate the functional role of L1 in neocortical development, we conducted in vivo L1 downregulation using the small hairpin RNA (shRNA) strategy and in utero electroporation (Young-Pearse et al.,^2007^). The shRNA plasmid targeting L1, which coexpressed monster green fluorescent protein (MGFP), was injected into the ventricular zone of the dorsal telencephalon of mouse embryos (C57BL/6J) at embryonic day 13.5, followed by electroporation. Neuronal migration and differentiation were then examined in histological sections 2 or 3 days after shRNA transfection. The results showed that downregulation of L1 perturbed neuronal migration, accompanied by alterations in the expression of some transcription factors in the cortical plate. These alterations might partially account for hydrocephalus, in which subcortical white matter and corpus callosum are hypoplastic, as seen in L1-KO mice.

## MATERIALS AND METHODS

### shRNA Constructs and Plasmid Vectors

**Fig 1:**
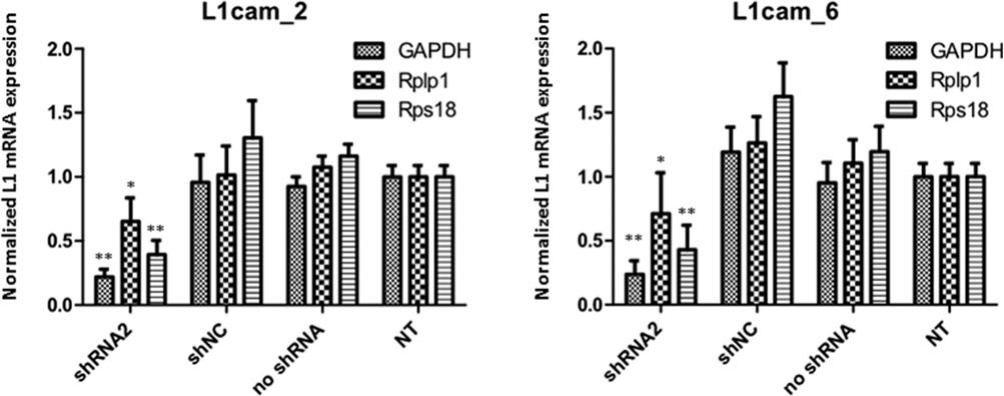
shRNA2 downregulated L1-mRNA in primary cultured mice cortical neurons. Primary cultured cortical neurons were transfected by shRNA2, shRNA-negative control (sh NC), and MGFP-only plasmid (no shRNA) using Amaxa Nucleofector. The expression level of L1 mRNA was significantly reduced in transfected cells with sh2 at 48 hr after transfection, compared with shNC and no shRNA as well as with no treatment (NT; ∗*P* < 0.05, ∗∗*P* < 0.01 by one-way ANOVA, sh2 vs. sh NC, no shRNA, and NT). We confirmed the expression level of L1-mRNA by using different primer sets, L1cam_2 and L1cam_6, and normalization with three housekeeping genes, *Gapdh*, *Rplp1*, and *Rps18*.

Linearized pGeneClip hMGFP Vector (Promega, Madison, WI) was used in this study. The small interfering RNA (siRNA) sequence targeting L1 was designed and provided by SuperArray Bioscience Corporation (Frederick, MD). We prepared four shRNAs targeting L1 cloned in pGeneClip hMGFP vector and a negative control shRNA (shNC) showing no homology to any of the known mammalian genes (i.e., having a scrambled sequence), as well as hMGFP-labeled mock plasmid (MGFP only plasmid). The siRNA sequences are shown in Table I. shRNA2 was used in the current study because it showed the highest level of L1 knockdown in vitro among the four plasmids examined (Supp. Info. Fig 1).

**Table I:** Targeting Sequence of siRNA Against L1cam

shRNA	Target sequence
shRNA1	CCACCTCAAGGGATACAATGT
shRNA2	AGCCTTACCAGAAGGGAAAGT
shRNA3	AGCCAATGCCTACATTTATGT
shRNA4	GACCCTGCAACTACTCAATGT
NCshRNA	GGAATCTCATTCGATGCATAC

### Cultures of Primary Cortical Neurons and Transfection

Dissociated cultures of cortical neurons were prepared from embryonic day 13.5 murine fetus (C57BL/6J purchased from CLEA Japan, Osaka, Japan). Briefly, the dorsal forebrain was dissected and treated in 0.125% trypsin/0.5 mM EDTA for 15 min at 37°C and then dissociated by gentle trituration (in a Pasteur pipette). In total 5 × 10^6^ dissociated cells were suspended in 100 μl of transfection reagent, and the cells were then transfected with 10 μg of shRNA using the Amaxa Nucleofector (Amaxa, Cologne, Germany), according to the manufacturer's instructions, using program No. O-005. We incubated the shRNA-transfected cells in 5 ml Neurobasal medium (Invitrogen Japan), supplemented with 2% B27 (Gibco, Invitrogen Japan), 0.5 mM L-glutamine (Gibco, Invitrogen Japan), and 1% penicillin/streptomycin solution (Nacalai Tesque, Tokyo, Japan). We added 4 ml of 1 × 10^6^/ml untransfected cells into the 1 ml shRNA-transfected cells (at a density of 1 × 10^6^/ml) and plated mixed cells at a final density of 1 × 10^6^ cells/ml in 24-well plates, which had been precoated with 0.1 mg/ml poly-D-lysine (PDL) and 50 μg/ml laminin (Invitrogen Japan) or 0.1 mg/ml PDL, 40 μg/ml anti-Fc antibody, and 1 μg/ml L1-Fc. All of the cultures were maintained at 37°C in a 95% air, 5% CO_2_ (v/v) humidified atmosphere. All animal studies were approved by the Institutional Review Board for Biomedical Research using Laboratory Animals at Kyoto Prefectural University of Medicine, and the animals were handled according to the institutional guidelines and regulations.

### Quantitative RT-PCR

Total RNA was extracted from the transfected cultured cells using an RNeasy Micro Kit (Qiagen Japan), and cDNAs were synthesized and amplified as described previously. Briefly, first-strand cDNAs were synthesized using an iScript cDNA Synthesis Kit (Bio-Rad Japan) with oligo(dT) primer. The relative gene expression levels of L1 were measured by qRT-PCR on an ABI Prism 7000 sequence detection system (Applied Biosystems Japan) using SYBR Premix Ex Taq (Perfect Real Time; Takara Bio Inc., Otsu, Japan) according to the manufacturer's protocol. The optimized number for thermal cycling was set at 40. The primers for L1 were designed as follows: L1cam_2: 5′-gctttgcctccgagggctgg-3′ (forward), 5′-ccagggacctgtactcgccga-3′ (reverse) and L1cam_6: 5′-ccaagtggagttccgctggacg-3′ (forward) and 5′-cttcggccacttgggggcac-3′ (reverse). TaqMan rodent control reagent (Applied Biosystems Japan) was employed for housekeeping genes, such as *Gapdh*, *Rplp1*, and *Rsp18*.

### Morphometry of Cultured Neurons

To evaluate the functional loss of L1 by shRNA targeting L1, at 48 or 96 hr after transfection, the primary cortical neurons were fixed with 4% paraformaldehyde for 5 min at 4°C. After fixation, we observed electroporated neurons expressing MGFP under a confocal microscope (FluoView FV1000 confocal microscope; Olympus, Tokyo, Japan). The length of the longest neurite, the number of branches, and the total length of the neurites were measured in NeuronJ, which is an ImageJ plugin allowing the tracing and quantification of elongated structures. Because the shRNA2-transfected cells showed a unique histogram with regard to the longest neurite extension at 48 hr after transfection, we classified the cells into three types, according to the longest neurite length: 1) neuron type 1, longest neurite less than 80 μm; 2) neuron type 2, longest neurite between 80 and 220 μm; and 3) neuron type 3, longest neurite more than 220 μm. All values are expressed as means ± SEM. The SEM values indicate the variation between mean values obtained from at least three independent experiments. Statistical comparisons were conducted by using Dunnett's multiple comparison test. *P* < 0.05 was designated as statistically significant.

### In Utero Electroporation

C57BL/6J pregnant mice were purchased from CLEA Japan Inc. (Osaka, Japan). The day on which the vaginal plug was found was designated as embryonic day 0 (E0). In utero electroporation was performed as described previously (Tabata and Nakajima,^2001^, ^2008^). Briefly, the pregnant mice were anesthetized with a mixture of ketamine and xylazine, and then the uterine horns were exposed. The expression vectors were diluted in phosphate-buffered saline (PBS) containing 0.05% trypan blue. The expression vectors (1 μl of 5 or 10 μg/μλ) were directly injected into the lateral ventricle of the telencephalon of E13 embryo with a glass micropipette. Thereafter, the head of the embryo was placed between the tweezers-type electrodes, which had disc electrodes of 5 mm in diameter at the tip. Five 50-msec 40-V electronic pulses were delivered at 950-msec intervals with an electroporater (CUY21SC electroporator; Nepa Gene Co., Ichikawa, Japan). After the electroporation procedure, the dams were sutured at the abdomen, and the pregnancy was allowed to continue. Embryos were harvested after 3 or 4 days, i.e., at E16 or E17.

### Immunohistochemistry

The fetal brains were dissected and fixed overnight with 4% paraformaldehyde, followed by cryoprotection in 20% sucrose at 4°C. Fixed brains were embedded in OCT compound and frozen in powdered dry ice. Serial coronal sections, at a thickness of 20 μm, were made using a cryomicrotome (Leica CM1850). After blocking with 10% goat serum, 1% bovine serum albumin (BSA), 0.01% Triton X-100 (Sigma-Aldrich Japan) in 0.1 M PBS, the sections were incubated in primary antibody at 4°C overnight; doublecortin (rabbit polyclonal, ab18723; Abcam, Cambridge, MA; 1:500), Satb2 (mouse monoclonal, ab51502; Abcam; 1:50), Ctip2 (rat monoclonal, ab18465; Abcam; 1:500), or Tbr1 (rabbit monoclonal, ab31940; Abcam; 1:500), diluted in 0.1% Triton X-100 and PBS. The sections were rinsed several times with PBS and then incubated in the secondary antibody (Alexa 647-conjugated goat anti-mouse IgG, Alexa 647-conjugated goat anti-rabbit IgG, Alexa 647-conjugated goat anti-rat IgG, 1:300, Invitrogen Japan). The sections were then coverslipped and observed under a confocal microscope (FluoView FV1000 confocal microscope; Olympus).

### Image Analysis

Images acquired with the Olympus FluoView FV1000 confocal microscope were processed with an Olympus FV10-ASW 1.7 viewer to adjust color and contrast. To conduct a quantitative analyses of the transfected cells, which were clearly detectable with MGFP fluorescence, at least three independent brains were analyzed at each embryonic stage: transfected at E13 and analyzed at E16 or transfected at E13 and analyzed at E17. The wall of the embryonic dorsal brain was divided into six layers, the ventricular/subventricular zone (VZ); the upper and lower intermediate zone (IZ); and the upper, middle, and lower cortical plate (CP; Fig. 4D). The way in which the IZ or the CP was divided into two or three subzones, respectively, was simply by dividing the region into two or three equal parts. The total numbers of MGFP-expressing transfected cells were counted in each of the six layers in each brain, and the ratio (percentage) of the cells present in each layer was calculated. We counted a total of 2,611 cells (n = 5), 4,809 cells (n = 5), 5,679 cells (n = 3), 4,163 c3lls (n = 5), 3,195 cells (n = 3), 3,150 cells (n = 3) from shNC (E13–16), shRNA2 (E13–16), MGFP-only plasmid (E13–16), shNC (E13–17), shRNA2 (E13–17), and MGFP-only plasmid (E13–17), respectively. We compared the ratios among brains transfected by L1 shRNA, negative control shRNA, and MGFP-only plasmid. Statistical analyses were performed with Kruskal-Wallis one-way analysis of variance and Dunn's multiple-comparisons test (SPSS 15.0 for Windows). The “cell process angle” was defined as the angle of the leading process of every labeled neuron relative to the tangential line at the subplate zone, specifically, the boundary zone between the IZ and the CP (see Fig. 5A,B). The measurements for neurons, which were migrating radially in the IZ toward the CP, were conducted with ImageJ software. These values were compared in SPSS 15.0 for Windows. With this program, Welch's two-sample *t*-test was performed to detect the differences in the dispersion of the “cell process angle.”

## RESULTS

### shRNA Downregulated L1 mRNA in Primary Cultured Cortical Neurons

**Fig 2:**
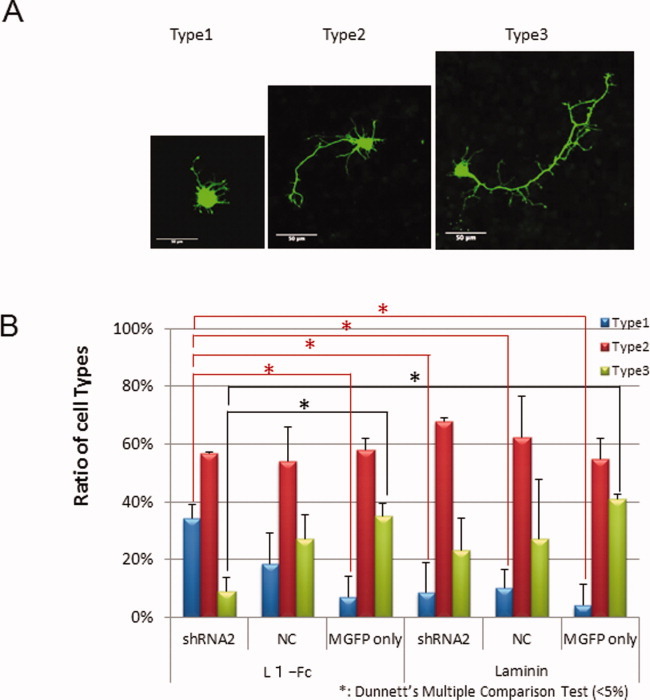
shRNA2 inhibited neurite outgrowth in primary cultured mice cortical neurons. A: Primary cultured cortical neurons transfected by shRNA2, shRNA-negative control (NC), and MGFP only plasmid showed three typical phenotypes, neuron type 1, type 2, and type 3, with the longest neurite length less than 80 μm, between 80 μm and 220 μm, and more than 220 μm, respectively. B: Ratio of transfected cells in each category. Type 1 neurons were significantly increased and type 3 neurons were significantly decreased in shRNA2-transfected cortical neurons compared with hMGFP-labeled mock plasmid (MGFP only)-transfected neurons when grown on L1-Fc substrate, as well as on laminin, whereas type 1 neurons were significantly increased in shRNA2-transfected cortical neurons grown on L1-Fc substrate, compared with those grown on laminin. ∗*P* < 0.05. Scale bar = 50 μm.

The expression level of L1 mRNA was significantly reduced in transfected cortical cells with shRNA2 plasmids at 48 hr after transfection compared with the negative control plasmid shRNA (shNC), no shRNA (MGFP only), as well as with no treatment (NT; *P < 0.05 by one-way ANOVA; Fig. 1). L1 mRNA expression was most decreased in shRNA2-transfected cells, and, accordingly, we used the shRNA2 plasmid in this study (Supp. Info. Fig. 1). Although shRNA2 induced a significant decrease in mRNA expression of L1 48 hr after transfection in primary cultured cortical neurons, it did not lead to a statistically significant decrease in L1 protein itself (Supp. Info. Fig. 2); however, this likely is due to a failure to achieve very high transfection efficiency.

### shRNA2-Induced Functional Loss in Primary Cortical Neurons

We examined the effect of shRNA targeting L1, using shRNA2, on primary cultured murine cerebral cortical neurons to determine whether it affected neurite outgrowth of the transfected neurons on an L1-Fc substrate. We compared the extent of neurite outgrowth on a glass dish precoated by either laminin/PDL or L1-Fc/PDL. The shRNA2 transfected cells showed a unique histogram with regard to neurite extension at 48 hr after transfection, which allowed us to classify cells into three subtypes with their longest neurites of different lengths, i.e., the type 1, type 2, and type 3 cells, with the longest neurite in each type being less than 80 μm, between 80 μm and 220 μm, and more than 220 μm, respectively (Fig. 2A). Forty-eight hours after transfection, the proportions of type 1, 2, and 3 cells showed no significant differences in shRNA2-, shNC-, and MGFP-only plasmid-transfected cells when grown on laminin substrate (Fig. 2B). On the other hand, when cells were grown on the L1-Fc substrate, the proportion of type 1 cell was 34% in shRNA2-transfected neurons and 9% in MGFP plasmid-transfected cells, which indicated a significant increase of the type 1 cells in shRNA2-transfected neurons. Similarly, type 3 cells accounted for 9% in shRNA2-transfected neurons, but 35% in MGFP plasmid-transfected cells on the L1-Fc substrate, which showed a significant difference. Furthermore, the percentage of type 1 cells was significantly increased in shRNA2-transfected neurons when cultured on L1-Fc substrate compared with those grown on laminin (Fig. 2B), which suggested that L1–L1 homophilic binding was disrupted in shRNA2-transfected neurons and that neurite growth, in general, was not disrupted by off-target effects, because neurite growth on laminin was not perturbed.

Ninety-six hours after transfection, type 1 and type 3 cells in shRNA2-transfected neurons made up 14% and 15%, respectively, and type 1 and type 3 cells in MGFP plasmid-transfected neurons made up 4% and 30%, respectively on L1-Fc substrate. This is the same tendency as was observed at 48 hr posttransfection, but it did not reach a statistically significant level.

### Downregulation of L1 Affected the Radial Migration in Murine Neocortex

**Fig 3:**
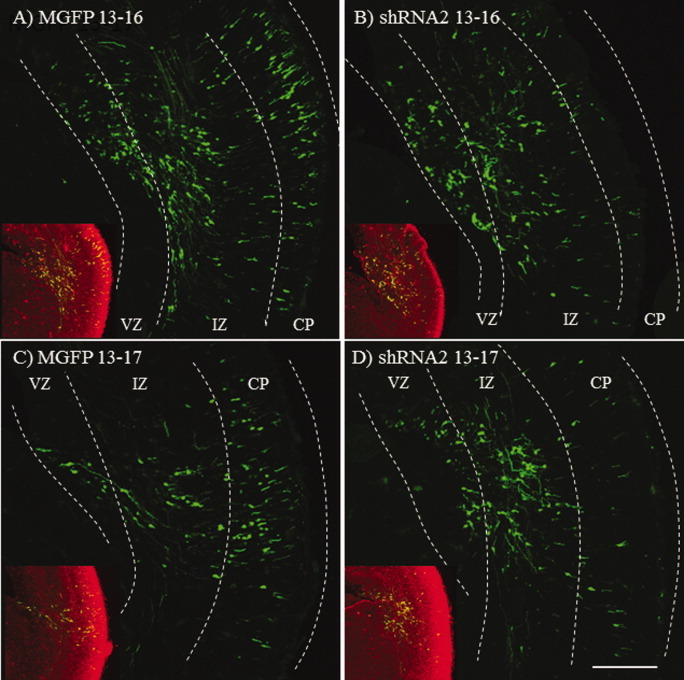
In utero electroporation of shRNA2 induced aberrant migration in locomotion and direction. A–D: In utero electroporation was performed at E13, and fetal telencephalon was observed at E16 (A,B) and E17 (C,D). A,C: MGFP only. B,D: shRNA2. In utero electroporation of shRNA2 induced decreased migration into the cortical plate and aberrant distribution and leading processes of transfected neurons in the intermediate zone. Scale bar = 200 μm.

**Fig 4:**
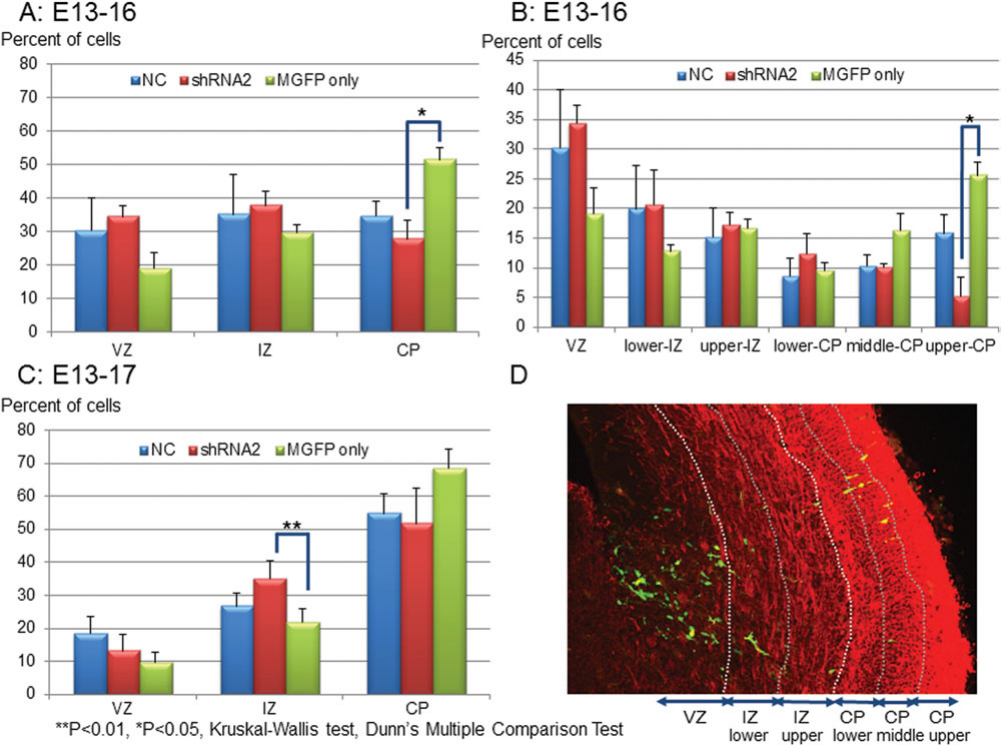
In utero electroporation of shRNA2 disrupted radial migration of cortical neurons. The percentage of transfected neurons in each layer, i.e., the ventricular/subventricular zone (VZ), intermediate zone (IZ), and cortical plate (CP), is shown. D: The dorsal telencephalon was divided into the six layers, as shown, used for morphometry. A,B: After in utero electroporation of shRNA2 at E13, shRNA2-transfected neurons showed a significant decrease in the CP at E16, especially in the upper CP. C: shRNA2-transfected neurons showed a significant increase in the IZ at E17. In shRNA2-transfected neurons, significantly fewer neurons migrated into the cortical plate, especially into the upper cortical plate at E16, and a significant number of neurons remained in the intermediate zone at E17. ∗*P* < 0.05, ∗∗*P* < 0.01.

To evaluate the function of L1 in cortical development, cortical progenitor cells in the VZ of the murine dorsal forebrain were transfected with the shRNA2, negative control shRNA (shNC), and MGFP-only plasmid by in utero electroporation at embryonic day 13 (E13). In utero electroporation allowed us to introduce shRNA into the fetal VZ to knock-down the targeted gene so that neuronal migration from the VZ toward the CP could be traced. We examined the distribution of MGFP-labeled cells in the presumptive somatosensory cortical area of the dorsolateral forebrain, which was located at the level of the medial and lateral ganglionic eminences by coronal section (Fig. 3). MGFP-only plasmid-transfected neurons possessed fasciculated leading processes in the IZ that extended straight, elongated neurites perpendicular to the marginal zone at E16, and almost 70% of those neurons had reached the CP at E17 (Fig. 3A,C). On the other hand, shRNA2-transfected neurons were scattered, showing disoriented and short leading processes in the IZ at E16 and small clusters of multipolar neurons with processes of a varied length and direction that remained in the IZ at E17 (Fig. 3B,D). We next performed morphometric analyses (Fig. 4), and 51% of cells transfected with MGFP-only plasmid had reached the CP at E16, whereas only 27% of shRNA2-transfected cells had migrated into the CP (*P* < 0.05; Fig. 4A). Moreover, the number of shRNA2-transfected neurons was significantly decreased in the upper CP, and 25% of MGFP plasmid-transfected cells had reached the upper CP at E16, whereas only 5% of shRNA2-transfected cells had reached that far (*P* < 0.01; Fig. 4B). At E17, 22% of the MGFP only plasmid-transfected cells existed in the IZ, whereas 34% of the shRNA2-transfected cells stayed in the IZ (*P* < 0.05; Fig. 4C). We did not find any significant differences in the position of migrating neurons between shRNA2 and the negative control shRNA plasmid. However, it should be taken into account that shRNA treatment itself generally induces off-target effects in vivo. In our experiments, this might have happened with negative control shRNA-transfected neurons in that they showed intermediate changes between shRNA2-transfected neurons and MGFP-only plasmid-transfected neurons. These analyses indicate that L1 knock-down by shRNA targeting L1 at E13 resulted in slowed migration of neocortical neurons.

### Downregulation of L1 Affects the Neurite Extension

**Fig 5:**
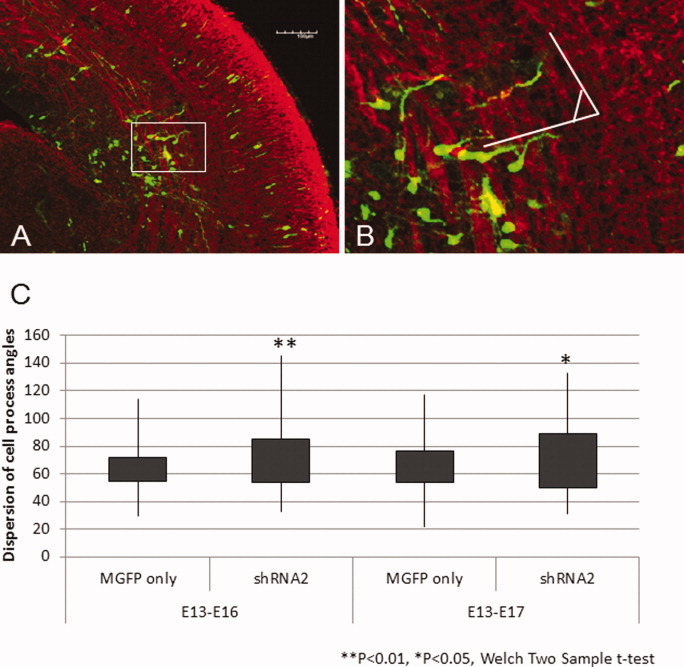
In utero electroporation of shRNA2 disrupted the direction of the radial migration of neocortical neurons. A,B: The angle of the leading process of the migrating neuron was defined as the angle of the extended process to the subplate zone. C: The angles of the leading process showed significant differences in shRNA2-transfected neurons at E16 as well as at E17. ∗*P* < 0.05, ∗∗*P* < 0.01.

When we observed the migration pattern of the GFP-expressing cells in the fetal telencephalon, we found that the cells transfected with MGFP-only plasmid showed a fine neurite fasciculation and radial migration to the marginal zone, similar to that found in the physiological situation. However, the neurons transfected with shRNA2 showed a wide variation in the orientation of neurites during migration. To confirm the difference in terms of the direction of cellular migration, we measured the angle of the leading neurite relative to the orientation of the CP, as described in Materials and Methods (Fig. 5A,B). Statistical differences in the angle of neurites were discerned between the cells transfected with shRNA2 and those with MGFP-only plasmid (Fig. 5C). The differences between the cells transfected with shRNA2 and those with shNC did not reach a statistically significant level (data not shown). These observations suggested that L1 downregulation disrupted correct orientation for radial neuronal migration during neocortical development.

### Alterations in the Expression of Transcription Factors Specific for the CP

To investigate whether the process of neuronal differentiation is affected by L1 knockdown, we studied the expression patterns of the transcription factors that are known to regulate the differentiation of cortical neurons, such as Satb2, Ctip2, and Tbr1. These factors are all expressed specifically and associated with the cortical layering. Satb2 (special AT-rich sequence binding protein 2) belongs to the family of DNA-binding proteins, which regulates gene expression through chromatin modification and interacts with other proteins. It is expressed in corticocortical projection neurons. Ctip2, belonging to the family of zinc finger transcription factors, is expressed in subcortical projection neurons (Leid et al.,^2004^; Arlotta et al.,^2005^; Chen et al.,^2008^; Leone et al.,^2008^). T-box brain 1 (Tbr1) is a member of a conserved protein family that shares a common DNA-binding domain, the T-box (Bedogni et al.,^2010^), and is found in the lower CP and subplate in normal development.

**Fig 6:**
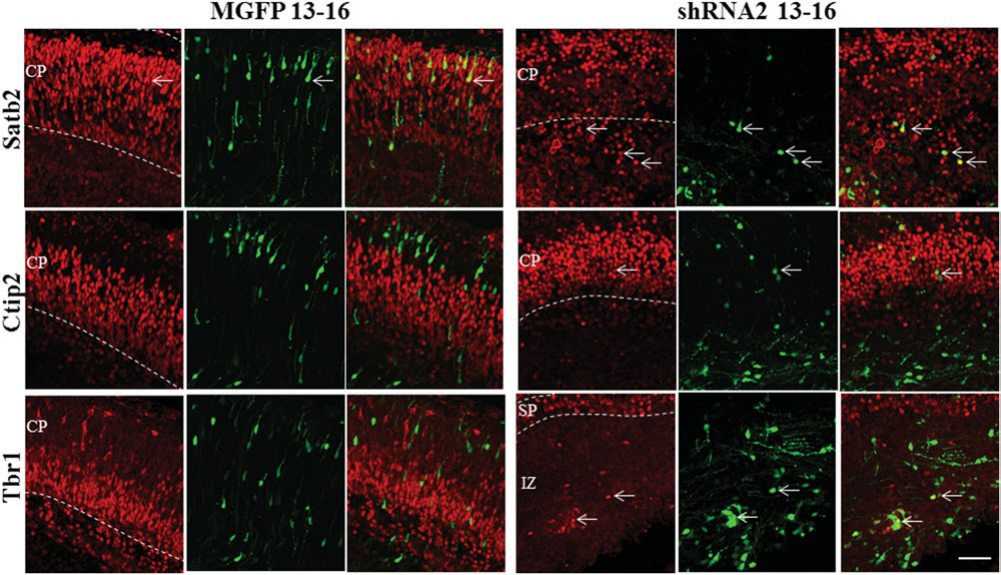
Aberrant expression of transcription factors specific for cortical layering was induced by in utero electroporation of shRNA2 at E16. Immunohistochemistry showed expression of the transcription factors (red) and transfected neurons (green). Related parts of the cortex, such as the cortical plate (CP), subplate (SP), or intermediate zone (IZ), are shown by by dashed lines. Many MGFP-only-transfected neurons reaching the upper and middle cortical plate showed intense immunoreactivity for Satb2 at E16, whereas many shRNA2-tranfected neurons migrating into the intermediate to subplate zones showed aberrant immunoreactivity for Satb2 at E16 (arrow). Many of the MGFP-only-transfected neurons that had reached the upper cortical plate showed no immunoreactivity for Ctips at E16, whereas only a very few shRNA2-tranfected neurons present in the lower cortical plate showed faint immunoreactivity for Ctip2 at E16 (arrows). Many MGFP-transfected neurons migrated into the upper cortical plate, showing no immunoreactivity for Tbr1 at E16, whereas many shRNA2-transfected neurons showed aberrant expression of Tbr1 in the intermediate zone (arrows) at E16. Scale bar = 50 μm.

Nontransfected neurons observed in both MGFP-only plasmid- and shRNA2-transfected dorsal telencephalon at E16 or E17 showed a similar immunoreactivity for these transcription factors. Satb2 expression was found from the upper to the middle portion of the CP and was expressed more weakly in the IZ. Many MGFP-only plasmid-transfected neurons migrated into the upper and middle CP, showing intense immunoreactivity for Satb2 at E16 as well as at E17, whereas shRNA2-tranfected neurons showed immunoreactivity for Satb2 mainly in the subplate and IZ at E16 and E17 (arrow, Fig. 6, Supp. Info. Fig. 3). Ctip2 immuoreactivity was observed in the lower half of the CP at the more rostral part of the cortex. Many MGFP-only plasmid-transfected neurons migrated into the upper CP, showing no immunoreactivity for Ctip2 at E16, and few expressed Ctip2 in the lower CP at E17, whereas very few shRNA2-tranfected neurons had migrated into the lower CP at E16 and E17, showing faint immunoreactivity for Ctip2 (arrow, Fig. 6, Supp. Info. Fig. 3). Immunoreactivity for Tbr1 was found in the lower CP and subplate. Many MGFP-only plasmid-transfected neurons had migrated into the upper CP, showing no immunoreactivity for Tbr1 at E16, and a few were expressed in the lower CP at E17. A few shRNA2-transfected neurons migrated into the lower CP, and aberrant expression of Tbr1 was observed in the IZ (arrow, Fig. 6, Supp. Info. Fig. 3) at E16. At E17, very few shRNA2-transfected neurons showed faint expression of Tbr1 in the lower CP.

## DISCUSSION

We found that the downregulation of L1 using in utero electroporation of shRNA affected the radial migration of cortical neurons, with aberrant expression of the transcription factors that are important for cortical layer formation. L1 plays a variety of functional roles required for normal brain development in different brain regions (Lemmon et al.,^1989^; Kamiguchi et al.,1998a). With regard to neuronal migration, however, only a very few reports have supported this concept (Lindner et al.,^1983^; Fischer et al.,^1986^). Although cerebellar granule cell migration in vitro was reported to be impaired by addition of anti-L1 antibody (Lindner et al.,^1983^), no studies on the role of L1 related to neuronal migration in the cerebral cortex have as yet been reported. There are two advantages to the methodology that we adopted to investigate the function of L1 expressed in neurons, compared with genomic knock-down. First, acute knock-down of L1 with shRNA in ventricular cells as well as migrating neurons in the developing murine telencephalon could exclude the compensatory mechanisms that might be recruited when L1 was knocked down in ES cells. Second, in L1-KO mice, all neurons in the brain lacked L1. In contrast, in our current experiments, only a subset of cortical neurons showed downregulation of L1, which generated a mosaic telencephalon in terms of L1 expression, so this method made it possible to study the dynamic movement of L1-downregulated neurons in a completely physiologically normal environment in vivo. This approach also suggests the L1 knockdown prevents the transfected cells from recognizing a guidance or migration cue. Global knockout would leave open the possibility that L1 is missing from the cells acting as a substrate.

Our observations suggested that downregulation of L1 induced either a delay in the exit of neurons from the VZ to the IZ or an inability to sense a molecular cue for neurons to migrate into the CP. Our study showed an increased ratio of multipolar shRNA2-tranfected neurons stuck in the IZ at E17, which partially resembled the migration disorder observed in doublecortin loss-of-function mice (LoTurco and Bai,^2006^), which showed an accumulation of multipolar-stage neurons in the IZ. It has been suggested that the locomotion of migrating neurons might be perturbed by L1 downregulation. It could be argued that the alterations in neuronal migration observed in the present study might have been caused by a loss of interactions among L1-downregulated neurons, normal cells, and extracellular ligands, because a lack of L1–L1 homophilic interaction or L1 heterophilic interactions with other CAMs, such as TAG-1/axonin-1, α1β3 integrin, neuropilin, or neurocan, might occur after the shRNA treatment (Ruppert et al.,^1995^; Montogomery et al.,^1996^; Yip et al.,^1998^; Kenwrick et al.,^2000^; Oleszewski et al.,^2000^; Silletti et al.,^2000^; Castellani et al,^2000^, ^2002^; Weller and Gartner,^2001^; Jacob et al.,^2002^).

We have demonstrated that downregulation of L1 perturbed not only the locomotion of neurons but also the orientation of the leading process. It is known that the intracellular domain of L1 contains an ankyrin-bindng region and a neuron-specific sequence, RSLE, which is critical for sorting L1 to axonal growth cones (Kamiguchi and Lemmon,^1998^). It is likely that the L1 downregulation perturbs pathfinding and the orientation of neurites, which leads to further deterioration of neuronal migration. To address this issue, a dynamic study using time-lapse videomicroscopy will be required.

To assess this phenomenon followed by downregulation of L1 and disturbed neuronal migration, we examined how the expression of transcription factors known to be involved in cortical development is affected. Satb2 is expressed in corticocortical projection neurons and plays essential roles in the axonal pathfinding of projection neurons in brain development (Britanova et al.,^2005^; Szemes et al.,^2006^; Alcamo et al.,^2008^; Chen et al.,^2008^; Leone et al.,^2008^). The onset of the expression of Satb2 was also observed in the IZ, prior to migration into the CP, correlating with the initiation of axon growth (Lickiss et al.,^2012^). It has been reported that the reduced size of the corpus callosum in the L1 knockout mice resulted from failure of the callosal axons to cross the midline (Demyanenko et al.,^1999^). Taking the functional role of Satb2 in the axonal pathfinding into account, it is tempting to speculate that the reduced expression of Satb2 in L1-downregulated neurons might be associated with the mechanisms underlying the hypoplasia or agenesis of the corpus callosum and hypoplastic subcortical white matter observed in L1-KO mice. Although Ctip2 is found in many brain regions, it is prominently expressed in subcortical projection neurons, and the Ctip2 gene mutation disrupts the corticospinal tract formation because of the failure of axonal growth into the spinal cord. It is speculated that the formation of the corticospinal pathway is affected in L1-downregulated neurons through delayed migration and aberrant expression of Ctip2, which is a phenotype observed in L1-KO mice. Tbr1 is a member of the T-box genes, which encode transcription factors involved in the regulation of a variety of developmental processes. Disruption of the mouse Tbr1 homolog demonstrated a critical role for Tbr1 in early cortical development. Our observation that a small number of the shRNA2-transfected neurons located in the IZ showed an aberrant expression of Tbr1 suggests that L1 downregulation might perturb the regulation of cell fate.

In conclusion, we have demonstrated that downregulation of L1 by using in utero electroporation of shRNA perturbed the locomotion and orientation of radially migrating neurons in neocortical histogenesis, together with alterations in the expression of transcription factors involved in cortical development. These results first indicate that L1 plays an important role not only in axonal guidance but also in neuronal migration during neocortical development. The methodological advantages of the in utero electroporation method we employed allowed us to downregulate L1 in only a subset of cortical cells, so that the novel functions of L1 in cortical development could be elucidated.
