# Supplementary figures and images for: Downregulation of L1 perturbs neuronal migration and alters the expression of transcription factors in murine neocortex
Source: J Neurosci Res. 2012 Oct 17;91(1):42–50. doi: 10.1002/jnr.23141 (PMC3533181; doi:10.1002/jnr.23141)

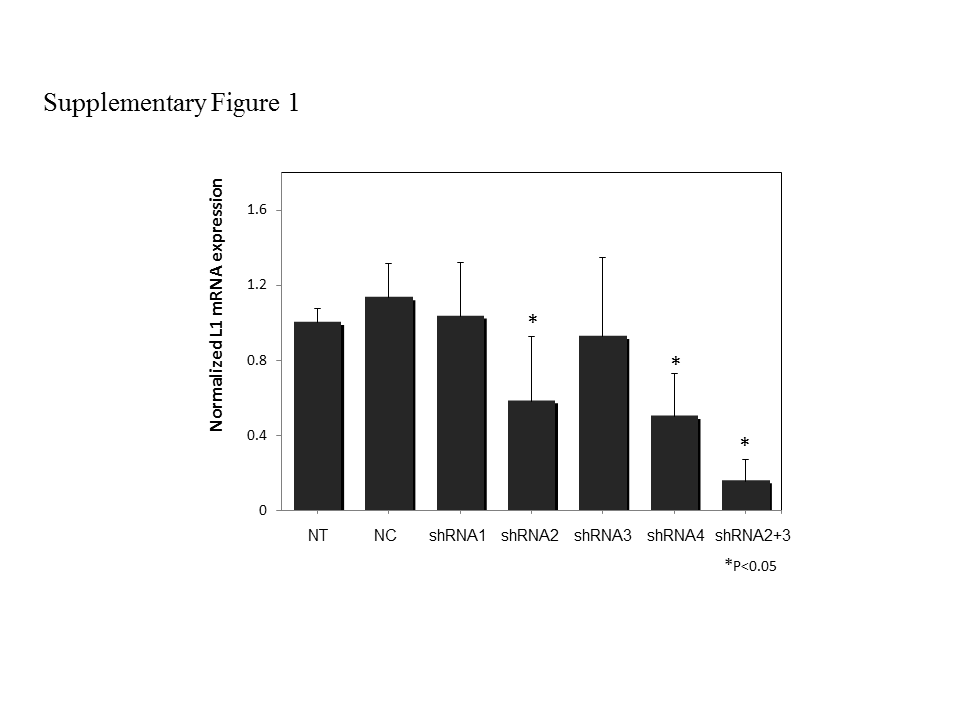

Supplement: Supplementary file 1 [file jnr0091-0042-SD1.tif]

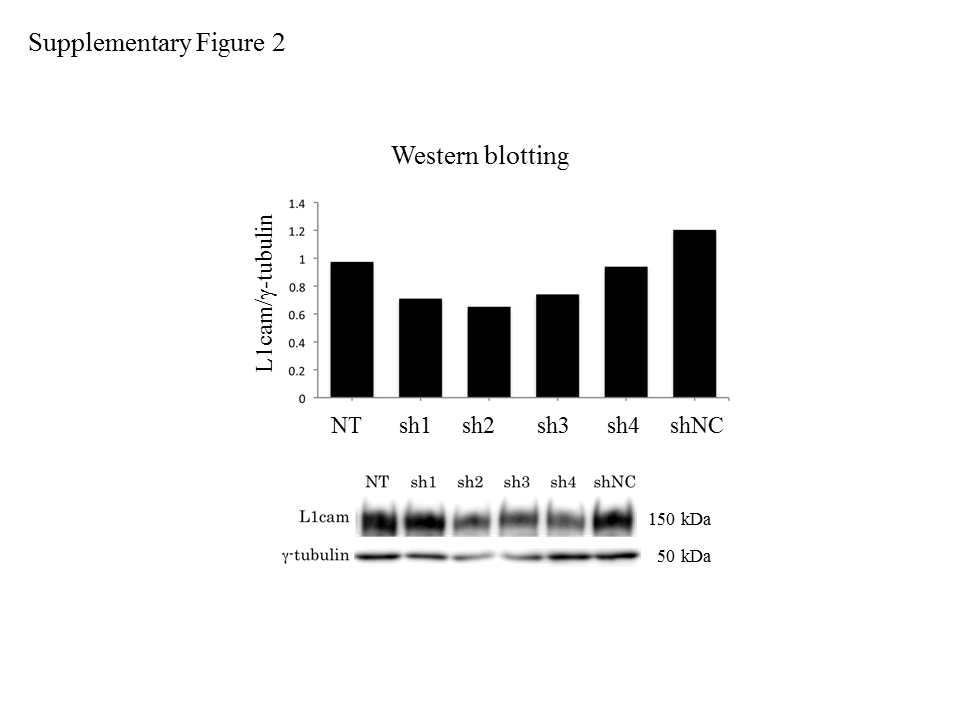

Supplement: Supplementary file 2 [file jnr0091-0042-SD2.tif]

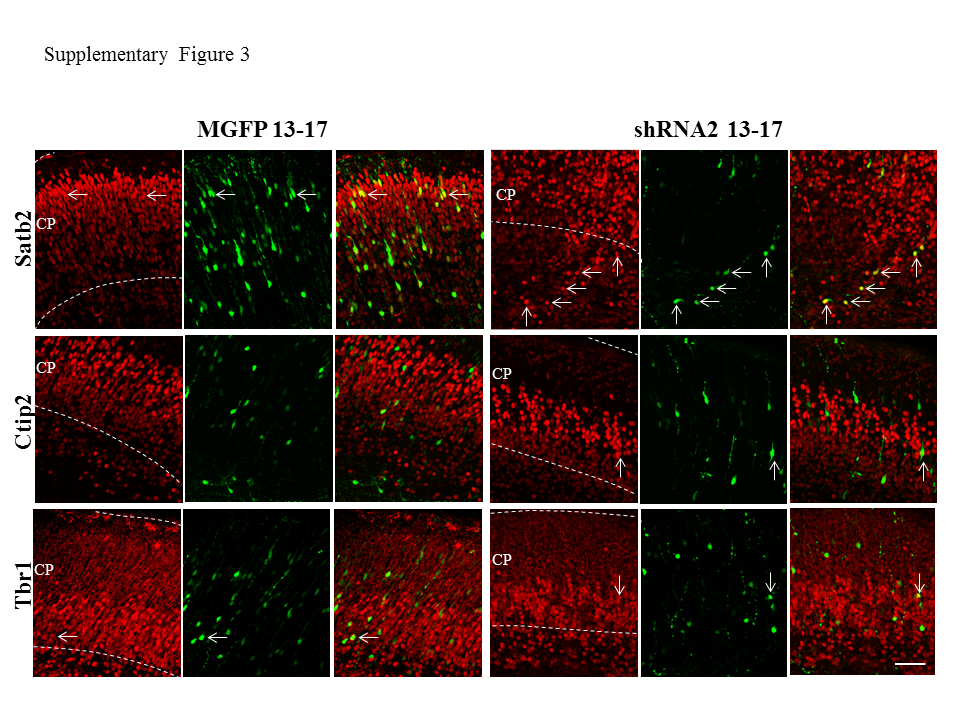

Supplement: Supplementary file 3 [file jnr0091-0042-SD3.tif]
