# Supplementary material for: Downregulation of L1 perturbs neuronal migration and alters the expression of transcription factors in murine neocortex
Source: J Neurosci Res. 2012 Oct 17;91(1):42–50. doi: 10.1002/jnr.23141 (PMC3533181; doi:10.1002/jnr.23141)
Supplement: Supplementary file 4 [file jnr0091-0042-SD4.RTF]

Supplementary data 1, 2, 3
Fig. 1. Normalized L1 mRNA expression in the neuroblastoma cell line. We tested the transfection efficiency of four shRNA plasmids targeting L1 which coexpressed MGFP fluorescence in a neuroblastoma cell line, Neuro2a cells, and found it was around 50&percnt; in every plasmid. The expression level of L1 mRNA was significantly reduced in transfected cells with shRNA2, shRNA2 &plus; shRNA3, shRNA4 plasmids at 48 hr after transfection, compared with the negative control plasmid shRNA (shNC), as well as compared with no treatment (*<I>P</I> &lt; 0.05 by one-way ANOVA).
Fig. 2. Protein expression of L1cam. Although L1 expression was most decreased in shRNA2-transfected cells at 96 hr after transfection by Western blotting, the difference did not reach the statistically significant level with Western blotting. We thus chose shRNA2 plasmid in the following study.
Fig. 3. Aberrant expression of transcription factors specific for cortical layering was induced by in utero electroporation of shRNA2 at E17. Immunohistochemistry showed expression of the transcription factors (red) and transfected neurons (green). The related part of the cortex, such as the cortical plate (CP), subplate (SP), and intermediate zone (IZ), was designated by dashed lines. Many MGFP-only-transfected neurons, reaching the upper and middle cortical plate, showed intense immunoreactivity for Satb2 at E17, while many shRNA2-tranfected neurons migrating into the intermediate to subplate zones showed aberrant immunoreactivity for Satb2 at E17 (arrow). Few MGFP only plasmid-transfected neurons expressed Ctip2 in the lower cortical plate at E17, whereas very few shRNA2-tranfected neurons migrating into the lower cortical plate showed faint immunoreactivity for Ctip2 at E17 (arrow). A few MGFP-transfected neurons and shRNA2-transfected neurons migrating in the lower cortical plate showed immunoreactivity for Tbr1 at E17 (arrow). Scale bar &equals; 50 &mgr;m.
